# Development of a Flexible Broadband Rayleigh Waves Comb Transducer with Nonequidistant Comb Interval for Defect Detection of Thick-Walled Pipelines

**DOI:** 10.3390/s18030752

**Published:** 2018-03-02

**Authors:** Huamin Zhao, Cunfu He, Lyu Yan, Haijun Zhang

**Affiliations:** College of Mechanical Engineering and Applied Electronics Technology, Beijing University of Technology, Beijing 100124, China; zhaohuamin909@163.com (H.Z.); hecunfu@bjut.edu.cn (C.H.); s201501113@emails.bjut.edu.cn (H.J.Z.)

**Keywords:** comb transducer, Rayleigh waves, broadband transducer, thick-walled pipes

## Abstract

It is necessary to develop a transducer that can quickly detect the inner and outer wall defects of thick-walled pipes, in order to ensure the safety of such pipes. In this paper, a flexible broadband Rayleigh-waves comb transducer based on PZT (lead zirconate titanate) for defect detection of thick-walled pipes is studied. The multiple resonant coupling theory is used to expand the transducer broadband and the FEA (Finite Element Analysis) method is used to optimize transducer array element parameters. Optimization results show that the best array element parameters of the transducer are when the transducer array element length is 30 mm, the thickness is 1.2 mm, the width of one end of is 1.5 mm, and the other end is 3 mm. Based on the optimization results, such a transducer was fabricated and its performance was tested. The test results were consistent with the finite-element simulation results, and the −3 dB bandwidth of the transducer reached 417 kHz. Transducer directivity test results show that the *Θ*_−3dB_ beam width was equal to 10 °, to meet the defect detection requirements. Finally, defects of thick-walled pipes were detected using the transducer. The results showed that the transducer could detect the inner and outer wall defects of thick-walled pipes within the bandwidth.

## 1. Introduction

As an important component that is subject to high temperatures and high pressures, thick-walled pipes have been widely used in the main steam pipelines of the electric power industry. However, the initial defects of the base metal of thick-walled pipes and the damage, defects and cracks occurring during the service process directly affect the service life of the entire structure of a thick-walled pipe and the operational safety of a power plant.

At present, mainly, thick-walled pipes are subject to random inspections using traditional nondestructive examinations, such as using magnetic particle testing, radiographic testing, and point-by-point ultrasonic testing, which are inefficient and have difficulty meeting the requirements of good engineering practices. Ultrasonic guided waves, used as a new nondestructive examination technique, can improve the detection efficiency for thick-walled pipes. Rayleigh waves, as one of the guided waves, can propagate for a long distance on the smooth surface of workpieces without reflection. It is especially suitable for defect detection on workpiece surfaces with Rayleigh waves, and has received extensive attention from scholars. Hassan and Babich et al. [1,2] studied the interaction between Rayleigh waves and semi-circular defects with a limited size on a workpiece surface through stimulating the Rayleigh waves with an angle probe. Thring [3] and Hernandez [4] characterized and detected surface defects by stimulating Rayleigh waves with EMAT (Electromagnetic Acoustic Transducer) and laser ultrasonic transducers, respectively. Yi et al. [5] studied the influence of the lift-off distance of the EMAT transducer on the cut-off frequency of Rayleigh waves during the process of nondestructive examination. Schaal [6] studied the transformation between Rayleigh waves and Lamb waves in delamination-like cracks.

Piezoelectric comb transducers are one of the most widely used transducers in guided wave nondestructive examinations, and are characterized by an easy operation, low cost, and high sensitivity [7,8,9]. Specific modes can be excited in the plates and pipelines by adjusting the space between piezoelectric elements and the width of the array element, controlling the input signal for phasing and time delay [10,11,12]. Rose et al. [13] studied the application of comb transducers for simulating guided waves for nondestructive examination by changing different parameters, both theoretically and experimentally. Yan and Quarr et al. [14,15] achieved the mode selection of piezoelectric annular array transducers under certain frequencies using an appropriate time delay and phase control. Glushkov et al. [16] achieved the mode selection of guided waves on a series of coaxial circular piezoelectric patches by applying periodic and replicated sinusoidal pulses. Kannajosyula et al. [17] obtained the minimum phase and delay value of the excitation signals when the modal guided waves were excited in the wavenumber domain, as well as corresponding relationships between the modal wavelength and piezoelectric ring width. Koduru [18,19] developed a circular PVDF (Polyvinylidene Fluoride) delay technique for modal excitation; Lamb waves were excited evenly to achieve defect detection in the plates. For the detection of defects in pipelines, Li [20] established an 8-channel time-delay system to control the excitation energy, time delay, and signal phase of different piezoelectric patches to increase the required modes and suppress unnecessary modes. Bareille et al. [21] arranged 16 pieces of piezoelectric material at one end of a pipeline, and excited the T mode using the shear vibration of piezoelectric patches, thereby detecting pipeline defects.

Hay et al. [22] coupled comb PVDF film to a pipeline through mechanical fixation, and then chose different wavelengths of modal guided waves, based on different electrode spacings to examine the pipeline. This method can be used to detect defects in different positions on inner and outer walls of pipelines that are filled with liquid or covered with a coating and insulating layer. Although PVDF piezoelectric transducers can achieve flexibility, their excitation performance is worse than that of PZT. Chang et al. [23] developed a flexible equidistant comb transducer using PZT and soft epoxy resin, so that the round hole of a polystyrene test block could be measured. In the same way, our research group applied the theory of mutual admittance combined with time-frequency analysis methods to study the detection of thick-walled pipe defects with flexible equidistant comb Rayleigh waves transducers. The array element spacing was equal to *λ*_R_ (wavelength of the surface wave), array element width was equal to *λ*_R_/2, and the number of array element *n* was equal to 5, which was the best parameter determined [24]. However, the above studies focused on narrowband transducers; the width and spacing of PZT needed to be adjusted to excite the guided waves of other modes, which was not efficient for the detection of various multi-sized defects. Therefore, a new flexible broadband Rayleigh wave comb transducer, based on PZT, was designed to detect the inner and outer wall defects of thick-walled pipes.

There are two commonly used ways to expand the bandwidth of a transducer. The first is to increase damping before and after the piezoelectric film to reduce electromechanical coupling coefficient *Q*_m_, so that the bandwidth is increased. The second is to utilize the multiple resonant frequency coupling characteristics of the piezoelectric materials. As long as two or more modalities of vibrations can be generated in a piezoelectric array element, or the higher-order resonant frequency and first-order resonant frequency spacing of one modality can be adjusted to make its combination frequency response not produce intermittent and deep trenches, its working frequency band will be broadened. The first method is very difficult to reduce *Q*_m_ to below 3, so it has limited effect on expanding the bandwidth of the transducer. Therefore, scholars have proposed a method of expanding the bandwidth of the transducer by using two or more mode couplings of piezoelectric materials, which has achieved good results [25,26,27].

**Figure 1:**
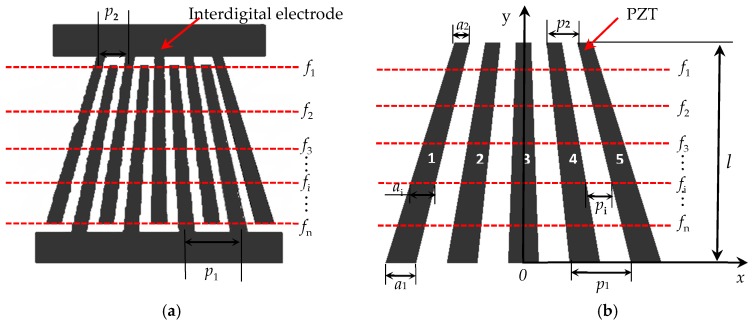
Non-equidistant comb transducer schematic diagram (**a**) Slanted Finger Interdigital Transducers (SFIT) (**b**) Non-equidistant comb transducer.

To achieve a broadband filtering effect, the traditional equidistant SAW (Surface Acoustic Wave) filter usually adopts the form of an inclined-type interdigital, known as SFIT (Slanted Finger Interdigital Transducers) [28,29]. The structure is shown in Figure 1a. Its interdigital electrode is in an inclined form, forming *n* frequency bands (*f*_n_) in the bandwidth interval, and each frequency band satisfies *p*_i_ = *c*_R_/*f*_i_,where *c*_R_ represents the velocity of the Rayleigh waves. The use of multiple frequency band coupling achieves a broadband filtering effect. In order to achieve the design goal of broadband operation, the comb transducer adopts a similar form. By adjusting the shape and size of the transducer array elements and using the nonrectangular array elements, multiple resonance frequencies of the array elements appear in the required frequency range and the broadband excitation of the transducer is realized through the coupling of multiple frequency bands. Transducer array elements shape are shown in Figure 1. The array elements are numbered 1–5 from left to right. Transducer array width and spacing gradually change from top to bottom. A Cartesian coordinate system is created with the center line of the 3rd element as the *y*-axis and the long bottom of the elements as the *x*-axis. The transducer array takes the *y*-axis as the axis of symmetry. The upper and lower margins of the trapezoid are *a*_1_ and *a*_2_, the spacings are *p*_1_ and *p*_2_, respectively, and the array length is *l*. The width and spacing of the element gradually change from the lower to the upper margins, corresponding to the lowest band and the highest band, respectively. Different array element widths correspond to different transducer band *f*_i_; similarly, arbitrary band meets *p*_i_ = *c*_R_/*f*_i_.

The band design goal of this broadband comb transducer is 500 kHz–1 MHz. When combined with the design conclusion of the equidistance transducer, *p*_1_ in Figure 1b corresponds to the wavelength of Rayleigh waves at 500 kHz and the upper base corresponds to the wavelength of the Rayleigh waves at 1 MHz, which shows that *p*_1_ = 6 mm, *a*_1_ = 3 mm, *p*_2_ = 3 mm, and *a*_2_ = 1.5 mm. The excitation performance and directivity of the transducer are determined by element length *l* and element thickness *t*. In what follows, the finite element method is combined with the multiple resonant coupling theory and will be used to optimize *l* and *t*.

## 2. Multiple Resonant Coupling Theory and Finite Element Optimized Broadband Transducer Array Element

### 2.1. Multimode Coupling Theory of Piezoelectric Materials

Different vibration modes will be generated according to the change of dimension parameters when a piezoelectric patch is polarized in the thickness direction and excitation voltage is applied to the thickness direction. Therefore, different resonant frequencies can be generated by adjusting the dimension parameters of the piezoelectric patch. When *l* and *w* >> *t*, the thickness stretching resonant frequency (*f_t_*) satisfy Formula (1) [30].

(1) ft=Ntt

where *N_t_* represents the thickness vibration frequency constant. For PZT5H, *N_t_* equals 2000 Hz·m.

**Figure 2:**
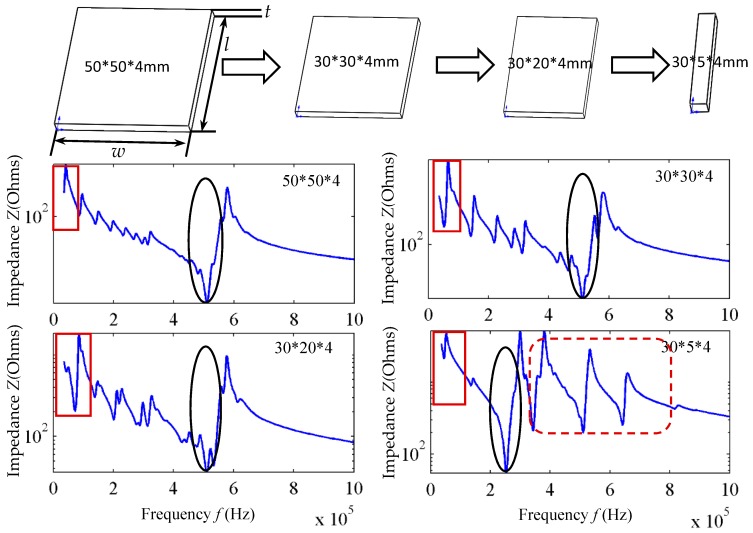
Analysis of resonant frequency of piezoelectric ceramics with dimension parameter change.

**Figure 3:**
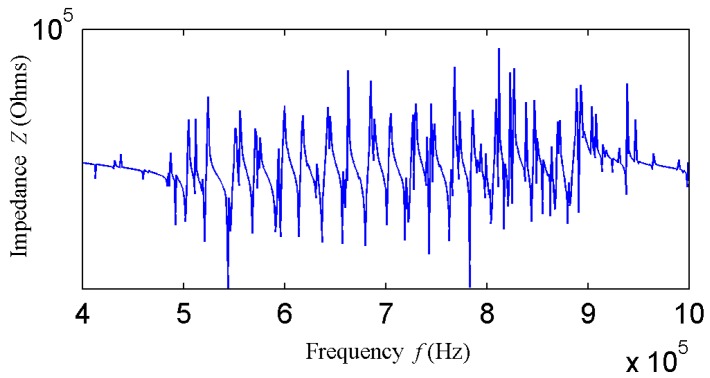
A schematic diagram of multiple resonant coupling theory.

As shown in Figure 2, when the elements *l*, *w*, and *t* are equal to 50 × 50 × 4 mm, 30 × 30 × 4 mm, and 32 × 20 × 4 mm, Formula (1) is basically satisfied, thus, the thickness-stretching resonance frequency is equal to 500 kHz. In Figure 2, the red boxes indicate the first-order length stretching resonant frequency of the piezoelectric patch; the round circles indicate the first-order thickness stretching resonant frequency, and the dashed boxes indicate the higher-order resonant frequency. As the *w* decreases from 50 mm to 5 mm, the element size parameters no longer meet *l*, *w* >> *t*, so the thickness-stretching resonant frequency decreases drastically, and coincides with the frequency range of the higher-order vibration modes. In view of this, dimensions of the transducer array element and the range of higher-order resonant frequencies can be adjusted, creating higher modes within the bandwidth required for the transducer; the first-order thickness resonant frequency and higher-order resonant frequency are coupled. Furthermore, as the transducer array transforms to an upper base of 1.5 mm and a lower base of 3 mm, more resonant frequencies will appear between 500 kHz and 1 MHz. The impedance curve of the trapezoidal array element is as shown in Figure 3; a number of continuous resonance frequencies appear between 500 kHz–1 MHz. Through the coupling of multiple resonance frequencies, broadband excitation of the transducer is achieved.

### 2.2. FEA Method to Optimize Array Element Length and Thickness

#### 2.2.1. Dimension Parameter Optimization of Array Element

According to the optimization results of the equidistant comb transducers, the thickness of the array elements is equal to 1 mm in the case of 500 kHz, and 1.2 mm in the case of 1 MHz, so three thickness dimensions of 1 mm, 1.2 mm and 1–1.2 mm were selected to conduct simulated optimization for a nonequidistance transducer. Element length *l* determines the sound field distribution and directivity of the transducer. The excitation sound field of the transducer has a near-field non-detection zone in the range of less than *l*^2^/4*λ* [30]; and, the non-detection zone is 75 mm at 1 MHz when *l* is equal to 30 mm. To avoid the non-detection zone being too large, the array element length cannot be too long. When the array element length is less than 15 mm, the transducer directivity is poor [24]. In view of this, three length dimensions of 15 mm, 20 mm, and 30 mm were studied. Three kinds of thickness and length were simulated by FEA via orthogonal experiments, and changes of their resonant frequencies were studied. While expanding the bandwidth of the transducer as the *Q*m of the transducer is reduced, the excitation performance of the transducer is also reduced. Therefore, the optimized result is to choose the parameter combination that makes the transducer bandwidth wider with the best excitation performance.

**Figure 4:**
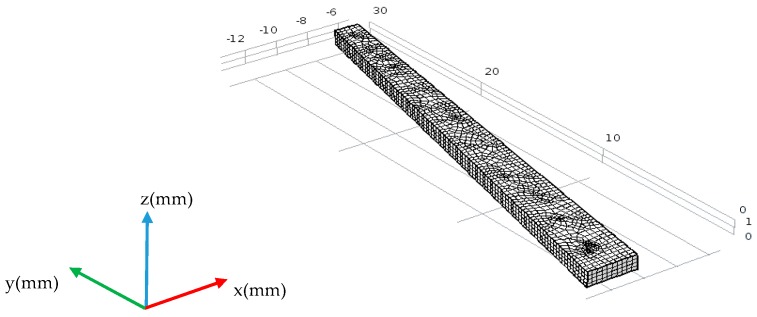
Frequency-domain-analysis simulation model of array element.

For frequency domain analysis, the model as shown in Figure 4 was established using the software COMSOL Multiphysics (5.2a, COMSOL INC., Stockholm, Sweden). The piezoelectric material is PZT5H; the frequency domain analysis ranges from 100 kHz to 1.2 MHz. The maximum size of the grid is 0.3 mm, and the minimum is 0.01 mm.

**Figure 5:**
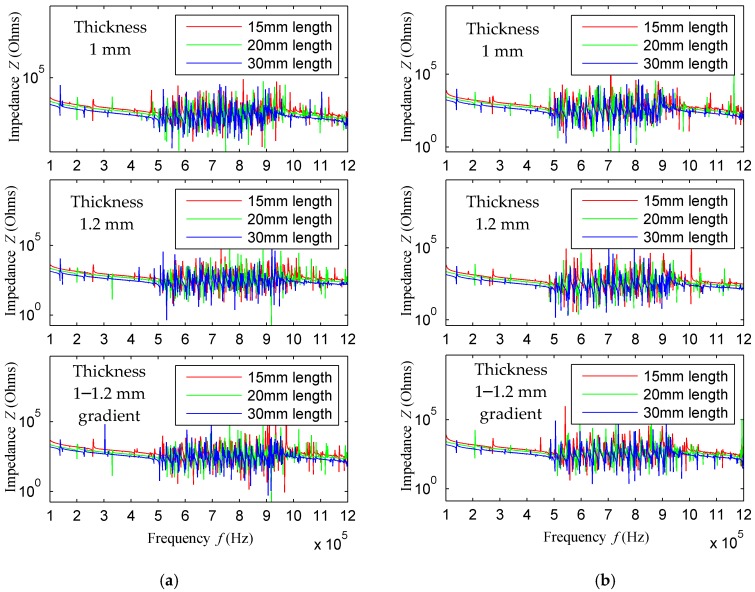
Impedance curves of array elements No. 1 and No. 2 with different array-element lengths and thicknesses: Impedance curves of (**a**) element No. 1 and (**b**) element No. 2.

The impedance curves of the piezoelectric patches with different thicknesses and lengths were obtained by finite element simulation. Shown in Figure 5a is the impedance curve of array element No. 1 in different dimension combinations. In the figure, when the element thickness is 1 mm, the impedance curves of the array elements of three lengths are compared. It is clear from the figure that the impedance value with the array element length of 30 is less than that of 20 mm and 15 mm, between 100–500 kHz and between 1–1.2 MHz. Although the impedance value is difficult to distinguish due to the existence of many resonant frequencies in the impedance curve between 500 kHz–1 MHz, from the impedance curves of the three element lengths at 100–500 kHz and 1–1.2 MHz, it can be concluded that when the element length is equal to 30 mm, the impedance value of the array element is less than that of 15 mm and 20 mm. Similarly, for the first array element, the conclusion is the same when the array element thickness is 1.2 mm and 1–1.2mm gradient. The same conclusion can also be drawn from Figure 5b for array element 2 and for elements 3, 4, and 5 that are not shown.

Therefore, the relationship between displacement *ξ* and impedance *Z* can be obtained by Formula (2) when the piezoelectric patch is vibrated along thickness [30]. As can be seen from Formula (2), the impedance *Z* is inversely proportional to the surface displacement *ξ*. The smaller the impedance of the piezoelectric patch is, then the larger the surface displacement and the better the excitation performance of the transducer will be. Therefore, the excitation performance of the transducer in the case of the array element length of 30 mm is better than that of 20 mm and 15 mm. As a result, the length of 30 mm is chosen as the length of array element.

(2) $$\xi =\frac{\left(1-k_{33}^{2}\frac{\tan(kt/2)}{kt/2})(nV-h_{33}D_{3}S\right)}{nZ}$$

where *C*_0_ represents the shunt capacitance of the piezoelectric patch; *ω* represents the frequency of excitation field; *k*_33_ represents the longitudinal electromechanical coupling coefficient; *V* represents the excitation voltage; *k* represents the number of stress waves generated in the piezoelectric patch; *h*_33_ represents the piezoelectric constant; *D*_3_ represents the point displacement in three directions of the three coordinate axes; *n* represents the electromechanical conversion coefficient; and, *S* represents the area of the piezoelectric patch.

**Figure 6:**
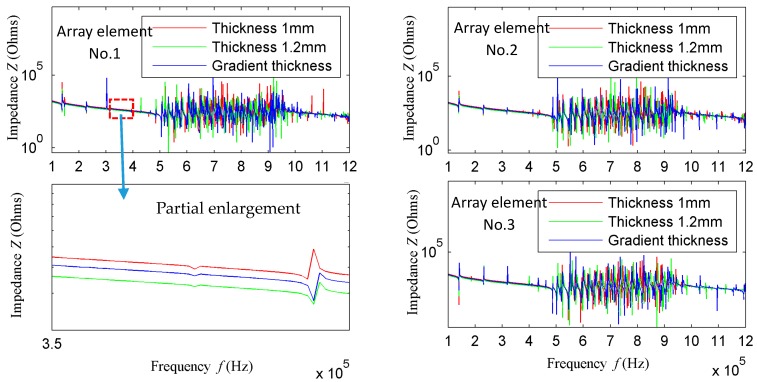
Impedance curves of different thicknesses of array element 1–3 when array-element length is 30 mm.

Figure 6 shows the impedance curves of different thickness of element 1, 2, and 3 when the element length is equal to 30 mm. As can be seen from the figure, the excitation bandwidth of the transducer meets the design requirements when the element length is equal to 30 mm. In addition, can be seen from the partial enlargement image when the element thickness is equal to 1.2 mm, the minimum impedance, largest surface displacement, and best excitation performance can be achieved, so the thickness of 1.2 mm is chosen as the element thickness.

#### 2.2.2. Study of Excitation Performance of the Transducer

**Figure 7:**
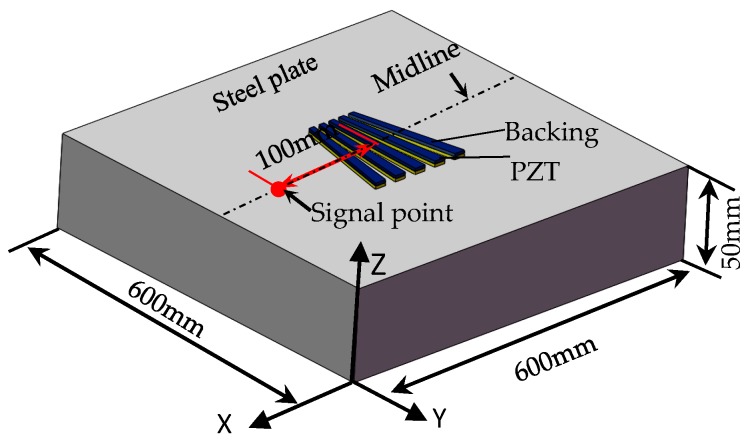
Simulation model of non-equidistant comb transducer.

In order to verify the selected thickness and length of the transducer, a model was established by PZflex finite element software (PZFlex, LLC 4.0, Weidlinger Associates Inc., New York City, NY, USA), and the time-domain analysis of the excited Rayleigh waves was carried out on the thick steel plate. Since thick-walled pipes have large diameters, the simulation results on the plate can be used to replace those of the thick-walled pipes, and this conclusion has been verified in the simulation of equidistant transducers. The three-diensional (3D) simulation model as shown in Figure 7 was established. According to the optimization results for equidistant transducers, signal tailing can be improved and bandwidth of the transducer can be increased by applying backing. In order to verify the excitation effect of the piezoelectric patches in different dimensions, the transducers with three kinds of thickness were designed, and backing was applied for simulation. The material parameters are shown in Table 1.

**Figure 8:**
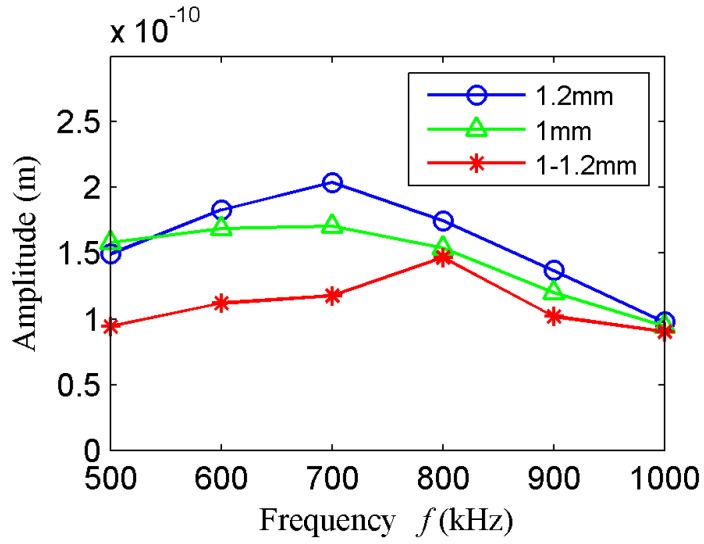
Rayleigh wave amplitude comparison of the comb transducer with different element thickness.

The steel plate is 600 mm wide and 50 mm thick; the left, right, and lower boundaries are absorbing boundaries. The upper surface of the plate is set as a free boundary. The boundary condition between PZT and steel is surface contact. The model mesh size is 20/*λ*_R_. A five-cycle Hanning window modulation sinusoidal signal is imposed on the five array elements of the transducer at the same time as the excitation signal, the excitation signals on the five elements have the same frequency and phase. The excitation frequencies are 500 kHz, 600 kHz, 700 kHz, 800 kHz, 900 kHz, and 1 MHz. Along the transducer center line in Figure 7, Rayleigh waves signal amplitude is taken at 100 mm away from the center of the transducer, then Figure 8 is derived.

The Rayleigh waves amplitude comparison of the comb transducer with different element thickness under different frequencies is shown in Figure 8 when the element length is equal to 30 mm. When the element thickness is equal to 1.2 mm, the Rayleigh waves amplitude is greater than that of 1 mm and thickness gradient. The conclusion is same as the analysis conclusion of array element impedance.

#### 2.2.3. Study of Directivity of the Transducer

**Figure 9:**
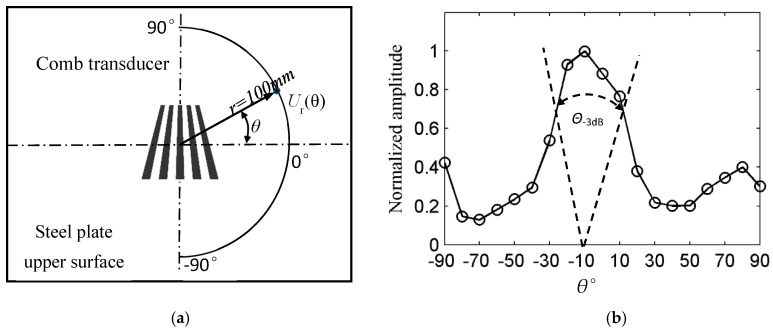
Finite element calculation of non-equidistant comb transducer directivity: (**a**) directivity research model; and, (**b**) Transducer directivity map.

Since the array elements of nonequidistant comb transducers are not parallel to each other, which will affect the directivity of the transducers. Directivity was studied in order to detect the defects better. As shown in Figure 9a, the Rayleigh waves signal amplitude was taken every 10° at *r* = 100 mm from the transducer center and then normalized. The transducer directivity diagram is as shown in Figure 9b. As can be seen from Figure 9b, the nonequidistant comb transducer directivity is deflected and the Rayleigh waves energy in the baseline line of the trapezium is larger than that of the upper, but the transducer has better directivity. *Θ*_−3dB_ beam width with 40° can meet the need of defect detection when the element length is equal to 30 mm.

## 3. Transducer Fabrication and Performance Test

### 3.1. Transducer Fabrication

**Figure 10:**
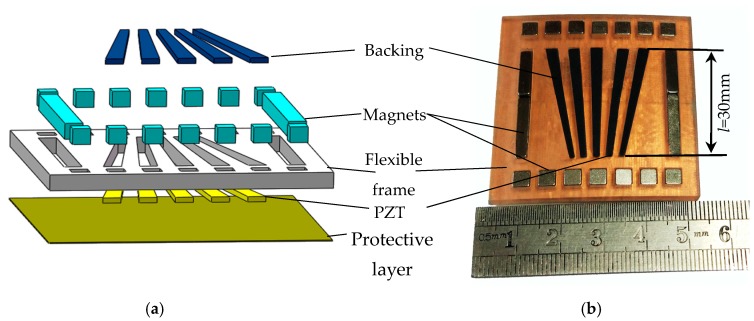
(**a**) Transducer three-dimensional (3D) analog decomposition diagram; (**b**) transducer physical map.

The non-equidistant comb transducer is fabricated according to the optimization results of finite elements, as shown in Figure 10a. The transducer consists of a backing, magnets, flexible frame, PZT, and protective layer. Firstly, the flexible frame is printed by a 3D printer, and the piezoelectric ceramic and fixed magnet holes are prepared on the frame. Since the transducer frame is flexible, several magnets are needed to ensure excellent coupling of all array elements on the pipeline surface. According to the simulation results of equidistant comb transducer, backing with a thickness of 3 mm can meet the requirement that the residual vibration of the transducer is absorbed. In view of this, a mixture of the 3 mm-thick epoxy resin with tungsten powder in a 4:1 ratio is used as the backing.

The manufacturing process of the transducer is as follows: firstly, place the flexible frame on the plane, and then place PZT in the frame; pour the mixture of tungsten powder and epoxy resin on the back of PZT. After the backing is solidified, the negative side of the piezoelectric patch is milled, and then the cathode is evaporated to ensure the same contact states of multiple PZT with pipelines. Finally, the protective layer is applied. The formed transducer is shown in Figure 10b.

### 3.2. Transducer Array Element Test

**Figure 11:**
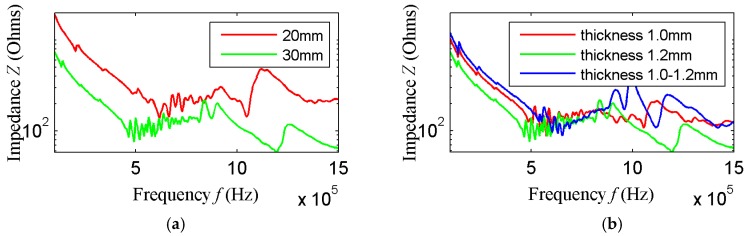
Impedance analysis experimental result of array element one; (**a**) Impedance analysis results of different lengths array element of thickness 1.2 mm; and (**b**) Impedance analysis results of length 30 mm array element with different thickness.

Piezoelectric patches with a length of 20 mm and 30 mm and a thickness of 1 mm, 1.2 mm and 1.2–1 mm were fabricated in order to verify the simulation results, and their impedances were analyzed using an Agilent4294A impedance analyzer (Agilent Technologies, Inc., Santa Clara, CA, USA). The frequency sweep results of array element 1 are shown in Figure 11a,b, indicating that the impedance of the piezoelectric patch with a length of 30 mm is less than that of 20 mm. Therefore, the excitation performance when the element length is equal to 30 mm is better than that of 20 mm, which is consistent with the simulation results. When the element length is 30 mm and the thickness is equal to 1.2 mm, the impedance is less than that when the thickness is 1 mm and is variable. The measurement results of other model elements are similar to those of array element 1, and are not shown. In Figure 11, the impedance curve between 500 kHz and 1 MHz changes more gently than in Figure 5 and Figure 6. The reasons may be: First, the collected data points are fewer when using the impedance analyzer, the resonance point of each resonant frequency cannot be fully scanned. Second, when the impedance analyzer performs the impedance analysis, the default voltage is 0.5 V, which is a low voltage, resulting in less vibration at some resonant frequencies, therefore, the impedance curve changes more gently.

### 3.3. Excitation Performance Test and Bandwidth Test of the Transducer

#### 3.3.1. Excitation Performance Test of the Transducer

In order to compare with the simulation results, two transducers in which array elements are 1.2 mm thick and 20 mm and 30 mm long are manufactured to test their excitation performance.

**Figure 12:**
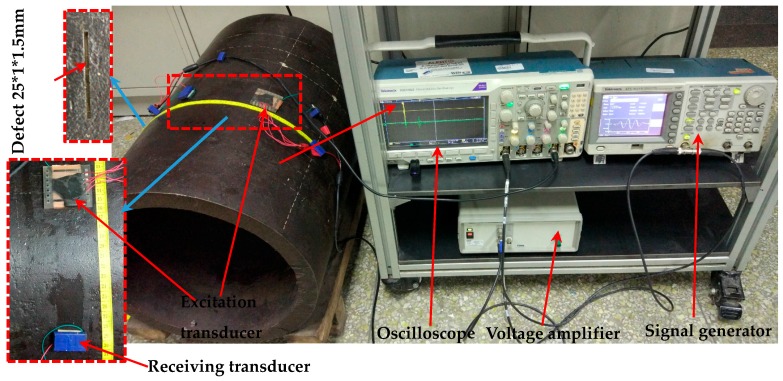
Thick-walled pipe defects testing equipment.

**Figure 13:**
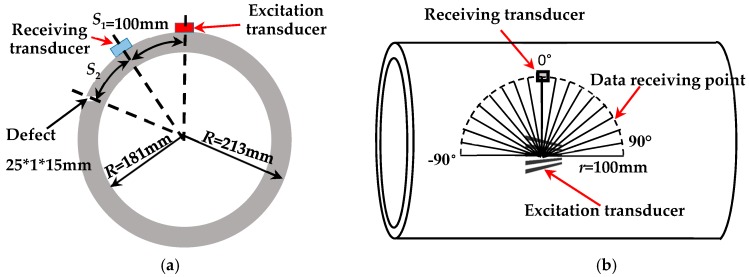
Test system diagram (**a**) Defect inspection test diagram; (**b**) Directivity test diagram.

The experimental setup is shown in Figure 12. The pipe is excited by a comb transducer and received by a single piezoelectric patch. The distance between the centers of the excitation transducer and the receiving transducer *S*_1_ is 100 mm. Test system diagram is shown in Figure 13a. The longitudinal wave velocity of the thick wall pipe is 5765.7 m/s, the shear wave velocity is 3195.2 m/s, and the density is 7800 kg/m^3^.

**Figure 14:**
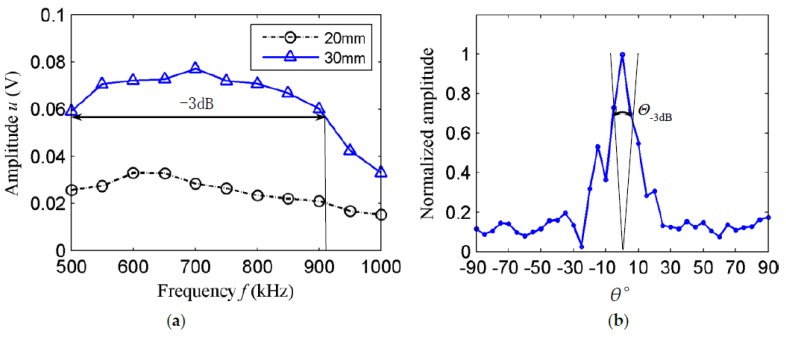
Transducer performance tests: (**a**) frequency-response test; (**b**) directivity test.

Excitation occurred at 50 kHz using 500 kHz–1 MHz signals, and the excitation Rayleigh waves amplitude of the two transducers is compared. As shown in Figure 14a, the transducer excitation Rayleigh waves amplitude of array element length 30 mm is greater than the transducer excitation Rayleigh waves amplitude of array element length 20 mm, which is consistent with the finite element calculation results. The test results also show that the −3 dB bandwidth of the transducer is 417 kHz, which meets the design requirements.

#### 3.3.2. Transducer Directivity Test

The directivity of the nonequidistant transducer with the element length of 30 mm was analyzed experimentally. The directivity test method is the same as the simulation, as shown in Figure 13b. The transducer directivity is shown in Figure 14b. As can be seen from Figure 14b, the excitation Rayleigh waves amplitude is subject to the angle of deflection; the Rayleigh waves amplitude within −40°–0° is greater than that of 0°–40°. Because the array element width is wider at −90°–0° and has a larger contact area with the pipe wall, the amplitude of the excitation Rayleigh waves is larger. However, the maximum value appears in the direction of 0°, which is slightly different from the simulation result. The reason for the discrepancy is as follows; comb transducer have the same coupling conditions for each array element when they are excited on the thick steel plate, so that the signal amplitude is greater on the −90°–0° side than on the 0°–90° side and its maximum appears at −10°. On the outer surface of the pipe, the axes of elements 1, 2, 4, and 5 are not parallel to the pipe axis. The coupling situations are different, the best coupling appears in the 0° direction, the coupling in other directions is weaker, so the maximum in the pipeline appears at 0°.

### 3.4. Defect Detection Experiment of Thick-Walled Pipe

The inner and outer wall cracks of a thick-walled pipe were detected using the non-equidistant comb transducer with an element length of 30 mm and an element thickness of 1.2 mm. An axial notch with an axial length of 25 mm, radial depth of 1.5 mm, and circumferential length of 1 mm was machined on the inner and outer walls of the thick-walled pipe by CNC EDM, in order to simulate the cracks. A non-equidistant comb transducer was used to verify defect detection capability. Experimental settings and photographs that are taken at the experimental site are shown in Figure 12 and Figure 13a. The transducer and the thick-walled pipe were coupled with glycerol. In the figure, *S*_1_ is the arc length between the excitation transducer and the receiving transducer, and *S*_2_ is the arc length between the receiving transducer and the defect.

#### 3.4.1. Detection of Cracks in Outer Wall of Thick-Walled Pipe

**Figure 15:**
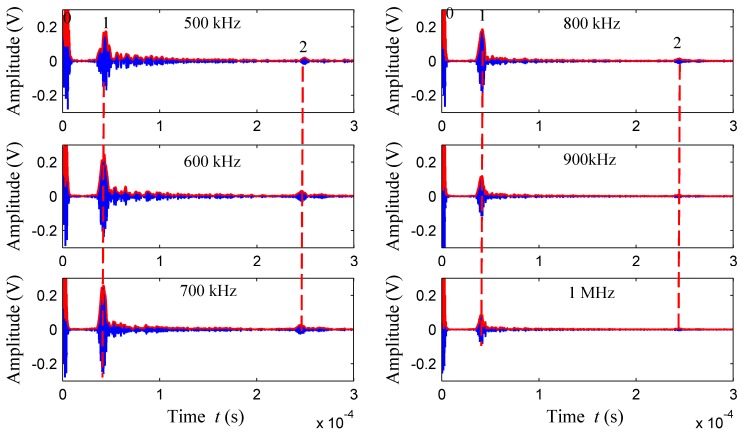
The time domain waveforms for crack defects of thick walled pipe outer wall.

*S*_1_ and *S*_2_ were defined as 300 mm, and the same defect was detected with 500 kHz, 600 kHz, 700 kHz, 800 kHz, 900 kHz, and 1 MHz excitation transducers. The defect detection capability of the nonequidistant comb transducers at different frequencies was tested. The time domain waveforms for crack defects are shown in Figure 15.

The Rayleigh waves velocity of the thick-walled pipe measured in advance was *v*_R_ = 2875.7m/s. Wave packet 0 refers to crosstalk signal, arrival time of wave packet 1 *t*_1_ = 0.379 × 10^−4^ s and wave packet 2 *t*_2_ = 2.423 × 10^−4^ s. After calculation, *L*_test_ = (*t*_2_ − *t*_1_) × *v*_R_/2 = 293.9 mm, which is close to the direct measurement *S*_2_ of the defect location, and the relative error is only (300 − 293.9)/300 = 0.02%. Therefore, it can be determined that the wave packet 2 is the defect echo. As can be seen from the figure, the defect reflection echo can be obtained in the time domain waveforms of 500 kHz–1 MHz. The time domain waveforms indicate that the non-equidistant comb transducer can get better echo signals at different frequencies and achieve good detection capability for outer wall cracks. As can be seen from the defect echo amplitude, defect detection capability of the transducer is related to the bandwidth, and it is consistent with Figure 13a. The best detection capability is within 600–800 kHz.

#### 3.4.2. Detection of Cracks in Inner Wall of Thick-Walled Pipe

**Figure 16:**
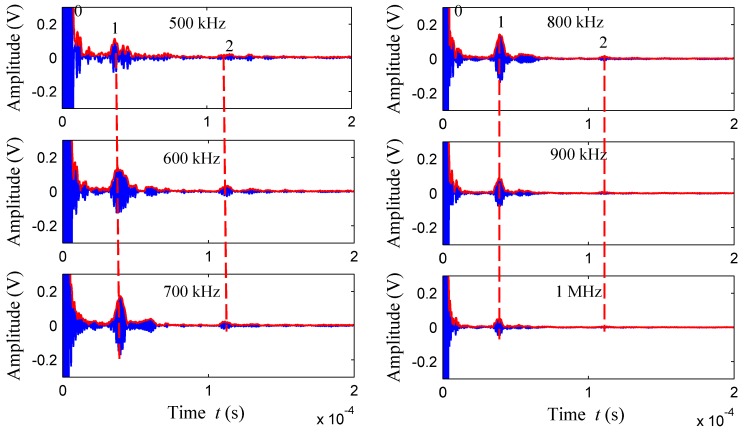
The time domain waveforms for crack defects of pipe inner wall.

The inner wall defects can be detected since the transducer is flexible, and the detection process is the same as the detection of outer wall defects. Firstly, *S*_1_ and *S*_2_ were defined as 100 mm, and Rayleigh waves at different frequencies were excited by non-equidistant comb transducer to detect cracks in the inner wall. The time domain waveform signal is shown in Figure 16. Through the time and wave velocity calculation, wave packet 1 is a direct wave, and wave packet 2 is the defect echo. The figure shows that the flaw echo signals are obvious within 500–900 kHz, and the echo signal cannot be received at 1 MHz, which is similar to the detection results of outer wall. The defect echo signal of the inner wall is smaller than that of the outer wall, which is caused by two factors. First, it is difficult for transducers to excite Rayleigh waves in the inner wall compared with the outer wall. Second, the inner wall is not as smooth as the outer, which affects the coupling of transducer and pipe.

### 3.5. Discussion

The above experimental results show that the transducer has good directivity and broadband characteristics, and can detect the cracks in the inner and outer walls of thick-walled pipes. However, some aspects need also to be improved.

(1) Due to problems in the transducer manufacturing process, they have relatively low excitation signal magnitudes. Therefore, the excitation performance of transducers needs to be further improved.
(2) The ability of such transducers in detecting defects in the outer walls of thick-walled pipe is better than in the inner wall; future transducer optimization should also focus on improving the detection of defects in the inner wall.

## 4. Conclusions

In this paper, a flexible broadband comb Rayleigh waves transducer with non-equidistant interval was designed using the finite element method combined with the multiple resonant coupling theory of piezoelectric ceramics. First, in the FEA orthogonal experiment, the optimal array element length and thickness are obtained by comparing the impedance values of array elements with different thickness and length. The array element length that isequal to 30 mm and thickness equal to 1.2 mm were found to be the best parameters. Then the FEA method and the experimental method were used to test the directivity and frequency response of the transducer. The experimental results showed that transducer directivity to meet the defect detection requirements and −3 dB bandwidth of the transducer were reached at 417 kHz; Defect detection experiments were also carried out. The detection of simulated cracks on the inner and outer surfaces of thick-walled pipes can be achieved between 600 kHz and 800 kHz, which provides a favorable solution for rapid defect detection in thick-walled pipes.

## Figures and Tables

**Table 1:** Material parameters for simulation.

Material	Density (kg/m^3^)	Longitudinal Wave Velocity (m/s)	Transverse Wave Velocity (m/s)
Low carbon steel	7900	5900	3200
PZT5H	7500	—	—
Backing	5710	1750	935
